# Screening of Bacteriocinogenic Lactic Acid Bacteria and Their Characterization as Potential Probiotics

**DOI:** 10.3390/microorganisms8030393

**Published:** 2020-03-11

**Authors:** Ana Pinto, Joana Barbosa, Helena Albano, Joana Isidro, Paula Teixeira

**Affiliations:** 1 Escola Superior de Biotecnologia, CBQF-Centro de Biotecnologia e Química Fina–Laboratório Associado, Universidade Católica Portuguesa, Rua Diogo Botelho 1327, 4169-005 Porto, Portugal; anapintomicro@gmail.com (A.P.); jbarbosa@porto.ucp.pt (J.B.); microhel@gmail.com (H.A.); 2 Bioinformatics Unit, Department of Infectious Diseases, National Institute of Health, 1649-016 Lisbon, Portugal; joana.isidro@insa.min-saude.pt

**Keywords:** antimicrobial activity, bacteriocin, lactic acid bacteria, probiotic

## Abstract

Probiotics are living microorganisms used as nutritional additives that confer health benefits on the host. Their use in food products is very attractive, especially if they could also inhibit important foodborne pathogens. In this study, antimicrobial activity against several foodborne pathogens was screened for 280 lactic acid bacteria (LAB) isolated from different food products and the probiotic characteristics of bacteriocinogenic isolates were evaluated. Seven out of 280 LAB isolates were selected due to their bacteriocinogenic properties and identified by 16S rRNA gene sequence analysis as *Pediococcus pentosaceus* (*n* = 6) and *Lactobacillus plantarum* (*n* = 1). Virulence factors and antibiotic resistances were not detected for any of the isolates. Except for *L. plantarum* R23, all the isolates were able to survive through the simulated gastrointestinal tract conditions. Only *P. pentosaceus* CFF4 was able to adhere to Caco-2 cells after the simulated gastrointestinal tract passage. In conclusion, even though in vivo studies should be performed, *P. pentosaceus* CFF4, which was also able to inhibit the growth of foodborne pathogens in vitro, seems to be a potential probiotic to be used in the food industry.

## 1. Introduction

Probiotics are defined as “live microorganisms that, when administered in sufficient amounts, confer health benefits on the host”, according to the Food and Agriculture Organization of the United Nations/World Health Organization [1].

Lactic acid bacteria (LAB) are considered the major group of probiotic bacteria, with *Lactobacillus*, *Lactococcus*, *Carnobacterium*, *Enterococcus*, *Streptococcus*, *Pediococcus*, *Propionibacterium* and *Leuconostoc* being the most common genera used [2,3]. Their ingestion as probiotics has been acknowledged to confer a range of health benefits, including the normalization of disturbed gut microbiota, prevention or alleviation of several intestinal disorders, prevention of heart diseases by lowering blood cholesterol levels, immune system stimulation, prevention of infectious diseases, among others [4,5]. Previous studies reported that probiotic bacteria may adhere and survive in the gastrointestinal (GI) tract in order to provide health benefits, where they act on the stability and protection of this ecosystem [6,7].

Most of the lactic acid bacteria/probiotics are ingested by the consumption of fermented foods, like dairy products, meat, vegetables and others [8]. Besides their use as biopreservatives, these microorganisms also enhance the texture and flavor of these kinds of products [9]. The levels associated with significant outcomes in clinical trials and found in probiotic products are in the range of 1–10 billion Colony Forming Units (CFU)/dose, which is within the recommended effective dose: more than 100 million CFU/dose [10,11]. However, food products containing potential probiotics should also meet the guidelines established by FAO/WHO [1]. In general, for commercial purposes, and depending on the product, potential probiotics that are species- or strain-dependent should also meet a number of requirements, including (i) safety: isolation from suitable habitats, screening and selection of probiotics in terms of phenotype and genotype pathogenicity, correct identification and antimicrobial susceptibility; (ii) functional: probiotics should be tolerant to the GI environment and possess intestinal epithelial adhesion properties; (iii) beneficial: lactic acid production and antagonism against pathogens; (iv) technology: tests for genetically stable strains are required for large-scale production and (v) physiological: immunomodulation, cholesterol metabolism, antimutagenic and anticarcinogenic properties are required assays [1].

A beneficial but non-mandatory criterion for the selection of probiotic strains may be the production of substances active against foodborne pathogens. The number of studies involving different bacteriocinogenic probiotic LAB that are active against foodborne pathogens—*Pediococcus pentosaceus* [9], *Pediococcus acidilactici* [12], *Lactobacillus plantarum* [13,14], *Lactobacillus lactis* [14] or *Enterococcus faecium* [15]—has been increasing continuously.

This study aimed to select different bacteriocinogenic LAB isolated from different food products and to evaluate their probiotic characteristics regarding safety, functional and physiological properties.

## 2. Materials and Methods

### 2.1. Microorganisms and Growth Conditions

In this study, 280 LAB isolates previously isolated from different food matrices (e.g., fermented meat sausages, fermented fish and vegetables) and belonging to the culture collection of *Escola Superior de Biotecnologia*, Porto, Portugal were characterized (Table S1). Each LAB isolate was grown on de Man, Rogosa and Sharpe (MRS) agar (Lab M, Bury, UK) at 37 °C for 48 h.

Thirty-two microorganisms were used as targets (Table 1) to test the inhibitory effects of LAB isolates. Each microorganism was grown on TSAYE—tryptic soy agar (TSA, Pronadisa, Madrid, Spain) with 6 g/L of yeast extract (YE, Lab M) at 37 °C for 24 h.

All microorganisms were stored at −20 °C in tryptic soy broth (TSB, Lab M) with 6 g/L of YE or MRS broth (Lab M) containing 30% (*v*/*v*) glycerol (Sigma, Steinheim, Germany), and sub-cultured twice before being used in assays.

### 2.2. Screening of Antimicrobial Activity of each LAB Isolate

Each target microorganism was grown in TSBYE (tryptic soy broth with 6 g/L of yeast extract) and spread on TSAYE and drops (10 μL) of each LAB culture (grown in MRS broth) were spotted on the lawns of targets and incubated overnight at 37 °C. Inhibition was recorded as positive if a translucent halo zone was observed around the spot [15].

To determine the nature of the inhibition for positive strains, each LAB suspension was centrifuged at 7000 rpm for 10 min at 4 °C (Hettich Zentrifugen, Rotina 35R, Tuttlinegen, Germany) and cell-free-supernatants (CFS) were adjusted to pH 6.0 with sterile NaOH (1 M) (Pronalab, Lisbon, Portugal; CFSn) and aliquots were treated for 1 h with 0.1 mg/mL of catalase (500 IU/mL, sterile; CFSnC) and 0.1 mg/mL of trypsin (CFSnCT), both from Sigma. After these treatments, each supernatant was spotted against target bacteria [15]. *Pediococcus acidilactici* HA-6111-2 was used as an anti-listeria control strain [17].

### 2.3. LAB Identification

#### 2.3.1. 16S rRNA Sequencing

Deoxyribonucleic acid (DNA) of LAB isolates with antimicrobial activity was extracted according to the protocol for total DNA purification from Gram-positive bacteria of the GRS genomic DNA kit-Bacteria -#GK07.0100 (Grisp, Porto, Portugal).

PCR amplification of the 16S rRNA gene fragments was performed using primers 27F (5′-AGAGTTTGATCCTGGCTCAG-3′) and 1492R (5′-GGTTACCTTGTTACGACTT-3′), as described by Vaz-Moreira et al. [18]. Products of PCR were purified with the GenElute PCR Clean-Up Kit (Sigma) and used as templates. Sequences obtained from an automatic DNA sequencer were subjected to BLAST analysis and similarities were determined using the National Center of Biotechnology Information databases (http://www.ncbi.nlm.nih.gov) [19]. The nucleotide sequences determined in the present study have been deposited in the GenBank database under the accession numbers MK999954–MK999960.

#### 2.3.2. Whole-Genome Sequencing

When 16S rRNA sequencing was not sufficient for species identification, whole-genome sequencing (WGS) was performed (2 × 150 bp) in an Illumina NovaSeq platform. Genome de novo assembly was performed with the INNUca v4.0.1 pipeline (https://github.com/B-UMMI/INNUca). Parsnp v1.2 was used for comparison with other publicly available (NCBI) genomes of the same genus with species inferred from the core genome phylogeny [20].

### 2.4. Safety Criteria of Potential Probiotics

All the tests described in Section 2.4. were performed in duplicate for each LAB isolate showing inhibition by the presence of proteinaceous compounds.

#### 2.4.1. Presence of Virulence Factors

##### Screening-Test of Biogenic Amines Production

This study was carried out according to the method developed by Bover-Cid and Holzapfel [21] for the detection of amino acid decarboxylase-positive microorganisms. The production of tyramine, histamine, putrescine, and cadaverine was assessed. Before the screening test, LAB isolates were sub-cultured seven times in MRS broth containing 0.1% of each precursor amino acid (all from Sigma): tyrosine-free base for tyramine, histidine monohydrochloride for histamine, ornithine monohydrochloride for putrescine and lysine monohydrochloride for cadaverine, and all were supplemented with 0.005% of piridoxal-5-phosphate.

Plates without amino acid were used as a negative control. A reaction was considered positive when a purple color appeared, or tyrosine precipitate disappeared around the colonies.

##### Production of Hydrolytic Enzymes

Gelatinase activity was assessed according to Tiago et al. [22] using the modified Luria–Bertani (MLB) broth supplemented with 50.0 g/L of gelatin. The presence of gelatinase was considered if the medium could no longer solidify at 4 °C.

DNase activity was tested as described by Ben Omar et al. [23] by using the medium DNase agar (Pronadisa) with 0.05 g/L of methyl green (Sigma). A clear halo around the colonies was indicative of a positive result.

The hemolytic activity was determined by streaking isolates on Columbia Agar plates with 5% defibrinated sheep blood (Oxoid, Hampshire, United Kingdom). Positive hemolytic activity was considered when the presence of clear halos around the colonies occurred (β-hemolysis) and negative hemolytic activity was considered when greenish zones (α-hemolysis) or the absence of clear zones (γ- hemolysis) around the colonies were observed.

*Staphylococcus aureus* ATCC 25213 was used as a positive control in all experiments.

##### Presence of Virulence Genes

The presence of virulence genes *agg* (aggregation substance), *gelE* (gelatinase), *esp* (enterococcal surface protein), *efaAfs* and *efaAfm* (cell wall adhesins) and *cylA*, *cylB*, *cylM*, *cylL_L_* and *cylL_S_* (cytolytic activity) was detected, as described by Barbosa et al. [24]. PCR amplifications were performed in a ThermoCycler (Bio-Rad, Richmond, CA, USA) in 0.2 mL reaction tubes each with 25 μL of mixtures using 0.5 mM of each primer, 0.1 mM of deoxynucleoside triphosphates (dNTP’s, ABGene, Surrey, United Kingdom), 1X of PCR Buffer (MBI Fermentas, Mundolsheim, France), 2.5 mM of MgCl_2_ (MBI Fermentas), 2U of Taq polymerase (MBI Fermentas) and 100 ng/μL of DNA (extracted as described in Section 2.3). The PCR program consisted of an initial denaturation step at 94 °C for 1 min, 35 cycles at 94 °C for 1 min, primer annealing at 55 °C for 1 min, 72 °C for 2 min, and a primer extension step at 72 °C for 7 min. After the last cycle, the products were cooled to 4 °C. The PCR products were analyzed by electrophoresis in 0.8% agarose gels with 1X TAE buffer (Bio-Rad). For each PCR reaction, a negative control (sample without template) and a positive control (sample with DNA from each strain according to the studied gene) were included.

#### 2.4.2. Antibiotic Resistance

The antibiotics used were ampicillin and chloramphenicol (Fluka, Steinheim, Germany), erythromycin, tetracycline and gentamicin (all from Labesfal, Tondela, Portugal), streptomycin and kanamycin (Sigma), as recommended by European Food Safety Authority [25]. The concentrations tested were based on the microbiological cut-off values established by the Panel on Additives and Products or Substances in Animal Feed (FEEDAP) of the EFSA [25], which allowed for distinguishing resistant from susceptible LAB isolates. Microbiological cut-off values (μg/mL) were determined by the agar dilution method, according to the Clinical and Laboratory Standards Institute [26]. All isolates were grown in Muller-Hinton Agar (MHA, BioMérieux, Marcy l’Etoile, France) with no added antibiotic as a negative control. The quality control strain *E. faecalis* ATCC 29212 was used to monitor the accuracy of MICs [26].

### 2.5. Functional Criteria of Potential Probiotics

#### 2.5.1. Inoculum

One colony of each LAB isolate (grown on MRS agar, 37 °C, 24 h) was transferred to 10 mL of MRS broth and incubated overnight at 37 °C. For the final inoculum, 0.1 mL of the last culture was transferred to 10 mL of fresh MRS broth and incubated in the same conditions. Cells were centrifuged at 7000 rpm for 10 min (Hettich Zentrifugen, Rotina 35R) and resuspended in 10 mL of sterile quarter strength Ringer’s solution to obtain an inoculum of approximately 10^7^ CFU/mL.

#### 2.5.2. Ability to Resist pH 2.5, pH 2.5 with Pepsin and Bile Salts

Survival of each LAB isolate was tested for different conditions in MRS broth: (i) adjusted to pH 2.5 (with 1M HCl), (ii) adjusted to pH 2.5 (with 1M HCl) and with the addition of 1000 U/mL of a sterilized solution of pepsin (Sigma) and, (iii) with no pH adjusted and with 0.3% (*w*/*v*) of bile salts (Pronadisa). For all these conditions, 1% of each inoculum, prepared as described above, was added to each solution. All the samples were kept at 37 °C and taken at time 0 (the time of inoculation) and every hour, until 4 h. Each experiment was done in duplicate and the growth of each inoculum in MRS broth was used as control. Serial decimal dilutions of each sample were made in sterile quarter strength Ringer’s solution (Lab M) and plated on MRS agar for enumeration by the drop count technique [27]. Microbial counts were transformed to logarithmic reduction using the equation: log (N/N0), where N is the microbial cell count after 4 h of exposure and N0 is the initial cell density.

#### 2.5.3. Resistance to Simulated Gastrointestinal Tract Conditions

The simulation of GI tract conditions was performed according to Barbosa et al. [28]. Aliquots of 0.5 mL of inoculum (prepared as described in Section 2.5.1) were placed into glass flasks with 49.5 mL of buffered peptone water (BPW; LabM, Lancashire, UK) adjusted to pH 2.5 with hydrochloric acid (1 M HCl) and with 1000 units/mL of a filter-sterilized solution of pepsin (Sigma). The glass flasks were kept at 37 °C and samples were taken at time 0 (time of inoculation) and every 30 min until a total of 60 min to simulate the conditions of the stomach. For the simulated conditions of the small intestine, a filter-sterilized solution of sodium hydroxide (1 M NaOH) was added to each glass flask in order to increase the pH from 2.5 to 7.0 and a sterile solution of bile salts (Pronadisa) was also added to achieve a final concentration of 0.3% (*w*/*v*). The flasks were held at 37 °C and samples were taken at time 0 (time of bile salts addition) and every 30 min for a total of 60 min.

Each experiment was done in duplicate and enumeration was performed as described in Section 2.5.2.

#### 2.5.4. Ability to Adhere to Human Colon Adenocarcinoma Cell Lines Caco-2

##### Preparation of Cell Lines Caco-2

The human colon adenocarcinoma cell lines Caco-2 (American Type Culture Collection ECACC 86010202) were cultured in Eagle’s minimal essential medium (Lonza, Verviers, Belgium) supplemented with 1% (*v*/*v*) of non-essential amino acids (Biosera, Boussens, France), 1% (*v*/*v*) of pyruvate (Lonza) and 20% (*v*/*v*) of fetal bovine serum (FBS; Biowest, Nuaillé, France) and were incubated at 37 °C and at 5% CO_2_–95% air atmosphere. The growth medium was replaced with fresh every second day.

##### Preparation of LAB

Cells of each LAB isolate, previously exposed to simulated GIT conditions, were centrifuged at 7000 rpm for 10 min at 4 °C (Hettich Zentrifugen, Rotina 35R), washed twice and resuspended in phosphate-buffered saline (PBS, 0.1M, pH 7.4; Sigma) to reach a level of approximately 10^9^ CFU/mL (volume of diluent was adjusted based on initial OD_600_ reading).

##### In Vitro Adherence Assays

Adhesion assays were performed with cells at late post-confluence (15 days in culture) and according to the method described by Guglielmotti et al. [29], with some modifications. In brief, 2 × 10^5^ Caco-2 cells/well were seeded in 24-well microplates and cultured in fresh EMEM media until appropriate confluence (90%) reached. Three wells of Caco-2 monolayers were inoculated with approximately 2 × 10^7^ of each LAB culture cells/well and the plates incubated at 37 °C with 5% CO_2_–5% air atmosphere. After 1 h of incubation, the monolayers were washed carefully three times with PBS and the Caco-2 cells were lysed with 1 mL of 0.2% (*v*/*v*) Triton-X 100 cold solution (Sigma) and vigorous pipetting. The Caco-2 cell lysates and respective LAB culture were serially diluted, plated on MRS agar and incubated at 37 °C for 72 h.

The colonies of all viable LAB cultures were counted and the CFU/mL calculated. Adhesion values (%) were calculated as follows: % Adhesion = (V1/V0) × 100, where V0 is the viable count of bacteria added initially and V1 is the viable bacteria count that adhered to Caco-2 cells, at the end of the experiment. Two independent assays were carried out. For each experiment, a positive control was performed with an adherent probiotic strain, *Lactobacillus rhamnosus* GG (ATCC), and wells without bacterial cells were used as a negative control.

### 2.6. Physiological Criteria of Potential Probiotics

#### Screening of the Isolates for Bile-Salt Hydrolase (BSH) Activity

Bile-salt hydrolase (BSH) activity was tested on MRS agar supplemented with 0.5% (*w*/*v*) sodium salt taurodeoxycholic acid or taurocholic acid (TCA) (Alfa aesar, Karlsruhe, Germany) and 0.37 g/L CaCl2. Sterile filter discs (6 mm) were placed on top of the culture medium and impregnated with 5 μL of each overnight LAB culture. As a negative control, 5 μL of MRS broth was used. All the plates were incubated anaerobically at 37 °C for 72 h. After incubation, the diameter of the precipitation zones was measured [30].

### 2.7. Statistical Analysis

An analysis of variance (one-way ANOVA) was performed for each isolate to test any significant effects of its exposure for 4h to different conditions (pH 7, pH 2.5, pH 2.5 with pepsin and bile salts). Also, differences between simulated gastrointestinal tract conditions on the survival of each isolate were assessed. Multiple comparisons were evaluated by Tukey’s post-hoc test and all analyses were performed using IBM SPSS Statistics, 25 (IBM Corporation, USA). The mean difference was considered significant at the 0.05 level.

## 3. Results and Discussion

### 3.1. Screening of Antimicrobial Activity of each LAB Isolate and Its Identification by 16S rRNA Sequencing

From 280 LAB isolates, 85 inhibited some target microorganisms by cell to cell competition, 31 inhibited by low pH and only seven inhibited by the presence of proteinaceous compounds—CFF4, CFF5, CFF51 and CFF202, all isolated from fermented fish [31]; R23, isolated from arugula; and Q42 and Q43, isolated from cheese. These isolates showed bacteriocinogenic activity against five out of the 31 target pathogens: *Bacillus subtilis*, *Listeria monocytogenes* 7946, *Listeria monocytogenes* 7947 and *Listeria innocua* 2030 C. *Enterococcus faecalis* ATCC 29212 was also inhibited by isolate R23. Thus, only these seven isolates were selected for further experiments and identified by 16S rRNA gene sequencing, which led to the identification of six *Pediococcus pentosaceus* (Q42, Q43, CFF4, CFF5, CFF51 and CFF202) and one *Lactobacillus plantarum* (R23). The identification of these isolates to the species level revealed that all presented 16S rRNA gene sequence similarity values higher than 99%. In Figure S1 is shown the phylogenetic relationship of the partial 16S rRNA gene sequences representing the isolates used in this study.

Since the species *L. plantarum*, *Lactobacillus pentosus* and *Lactobacillus paraplantarum* are genotypically related, the 16S rDNA sequencing alone is not sufficient to conclude that isolate R23 belonged to *L. plantarum* species [32]. Thus, its total genome was sequenced, which allowed us to confirm its identification as *L. plantarum* and the reads of *L. plantarum* R23 genome sequence were submitted to the European Nucleotide Archive—ENA; https://www.ebi.ac.uk/ena—provided from EMBL-EBI, under Bioproject Accession number: PRJEB32816 and Sample Accession number: ERS3473554).

Many strains belonging to the LAB group are extensively isolated from a great variety of fermented products since, besides having many improved nutritional and technological features, they also have generally recognized as safe (GRAS) status. In addition, a beneficial feature is the production of antimicrobial substances that allow the prevention of foodborne pathogens and bacteriocinogenic activity of several LAB against foodborne pathogens such as *Listeria monocytogenes*, *Bacillus cereus* or *Staphylococcus aureus* have continuously been reported [9,13,15,33,34].

Worldwide, there is an increasing consumer demand for healthy, chemical preservatives-free and safe products.

Due to this high demand, the number of studies trying to prove the potential of LAB isolates as probiotics has also been increasing [15,35,36,37].

### 3.2. In Vitro Screening of Probiotic Properties of Selected LAB

Biogenic amines and hydrolytic enzymes were not produced by the seven LAB studied, as well as none of the virulence genes tested. To be considered as probiotic, the bacterium must be free of virulence determinants in order to ensure that it will not cause any harm to the consumers. Other authors have also found other LAB isolates, such as *Pediococcus* and *Lactobacillus*, to be absent of virulent determinants [16,38,39].

Candidate probiotic bacteria should not act as reservoirs for antibiotic resistance genes [40]. According to the microbiological cut-off values defined by FEEDAP Panel (Table 1 in [25]), all LAB isolates were inhibited at concentrations equal to or lower than the established cut-off value for all antibiotics tested, i.e., no antibiotic resistances were observed in all isolates (Table 2). Regarding antibiotic resistances and according to FEEDAP Panel, these LAB isolates are considered as acceptable and may be used as a feed additive [25]. Furthermore, since the selected isolates belong to genera *Lactobacillus* and *Pediococcus*, they are included in the list of QPS *status* (Qualified Presumption of Safety) determined by the Food Safety Authority [41,42].

**Figure 1:**
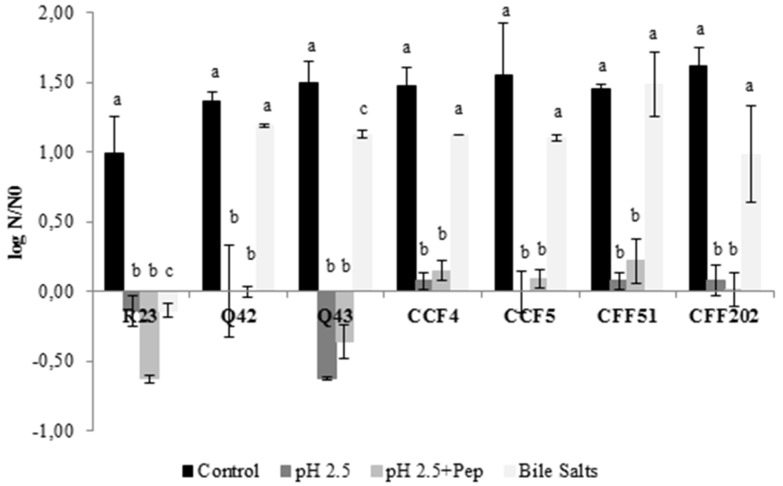
Effect of exposure to different conditions for 4 h on the survival of seven LAB isolates. Logarithmic reduction in comparison with the initial cell concentration. The results are means based on data from two replicates and standard deviations are indicated by error bars. Equivalent lowercase letters, per isolate, mean no significant differences between each condition (*p* > 0.05).

As already stated, probiotics should resist several conditions and remain viable to exert health benefits on the host. Logarithmic reduction to each exposed condition is presented in Figure 1, for all LAB isolates. All isolates showed good tolerance to bile salts after 4 h compared to the control, with significant differences (*p* < 0.05) obtained only for isolates *L. plantarum* R23 and *P. pentosaceus* Q43. Regarding the exposure to acidic conditions and acidic condition with pepsin, no significant differences were obtained between both conditions (*p* > 0.05) and only isolates *L. plantarum* R23 (~0.6 log cycles) and *P. pentosaceus* Q43 (~0.5 log cycles) showed a slight reduction. Despite these, no reduction exceeded 1 log cycle, meaning that it is possible to assume that these isolates, even *L. plantarum* R23 and *P. pentosaceus* Q43, remained viable. Several studies showed similar results in the screening of probiotic properties [43,44].

To guarantee that potential probiotics were able to survive through the GI tract, each LAB isolate was exposed to simulated sequential GI tract conditions. The results obtained are shown in Table 3. During the exposure to conditions of the stomach (pH 2.5 with pepsin) for 60 min, significant differences (*p* < 0.05) were only obtained for *L. plantarum* R23 which exhibited a reduction of 2.4 log cycles. Isolates *P. pentosaceus* Q42, CFF4, CFF5 and CFF202 were also reduced, but with lower log reductions, (<0.5 log CFU/mL). After subsequent exposure to bile salts, i.e., to conditions of the small intestine, slight decreases were also recorded for all LAB, with no significant differences observed (*p* < 0.05). This means that after GI tract passage, all LAB (except *L. plantarum* R23) were able to survive, maintaining their initial number (about 10^7^ CFU/mL). Other authors have reported the ability of different strains of *P. pentosaceus* to tolerate the GI tract conditions [9,45,46]. However, it is important to highlight that these experiments were done without the protection of a food matrix and in a real situation the behavior of the sensitive *L. plantarum* R23 could be different. Barbosa et al. [15] reported that an *E. faecium* strain, which was reduced to values below the detection limit of the enumeration technique by the end of GI passage simulated in BPW, showed a reduction of only ca. one log cycle by the end of the same conditions simulated in *alheira* food matrix.

After exposure to GI tract conditions, none of the studied isolates was able to adhere to Caco-2 cells, with the exception of *P. pentosaceus* CFF4, for which 11% adhesion was observed. Adhesion of LAB is a complex process involving contact between the bacterial cell membrane and interacting surfaces and is strain-specific [47], which could explain the different behaviors among the six *P. pentosaceus* isolates. The same was observed for different *P. pentosaceus* strains studied by other authors; Damodharan et al. [45] found high adherence (~34%) of *P. pentosaceus* KID7 to Caco-2 cells, while Han et al. [46] found similar results (~11%) for *P. pentosaceus* R1. The ability to adhere can provide information about the ability of candidate probiotics to colonize and may modulate the host immune system. Therefore, only *P. pentosaceus* CFF4 achieved this prerequisite.

Bile-salt hydrolase activity was not found for the seven LAB isolates tested. Tsai et al. [30] also screened the BSH activity by 800 LAB strains and only found 22 with positive results. The microbial activity of BSH in the host results in the reduction in cholesterol levels, which despite being desirable, is not an essential characteristic of probiotics [48,49].

## 4. Conclusions

Among the 280 LAB isolates screened, only seven (six identified as *P. pentosaceus* and one as *L. plantarum*) showed bacteriocinogenic activity and were selected for further studies. All the isolates lacked virulence determinants or antibiotic resistance. They were also able to survive through the simulated GI tract conditions, except for *L. plantarum* R23, which was reduced by more than two log cycles at the end of the small intestine conditions. *Pediococcus pentosaceus* CFF4 was the only isolate able to adhere to Caco-2 cells (11% of adherence after the simulated GI tract passage).

This study demonstrated that at least one isolate, *P. pentosaceus* CFF4, presented characteristics of a potential probiotic and also inhibited the growth of foodborne pathogens. Even though this probiotic candidate complies with the QPS standards of EFSA and is considered safe, additional tests should be performed to validate in vivo the potential of this strain.

## Figures and Tables

**Table 1:** Microbial strains used in this study.

Microorganisms	Species	Source
Gram-positive	*Bacillus cereus* *Bacillus subtilis* *Bacillus stearothermophilus* *Listeria innocua* 2030c *Staphylococcus aureus* 18N (methicillin-resistant > *Staphylococcus aureus-*MRSA) *Staphylococcus aureus* 2037 M1 (methicillin- sensitive *Staphylococcus aureus-*MSSA)	ESB culture collection
	*Enterococcus faecalis* ATCC 29212 *Staphylococcus aureus* ATCC 29213	ATCC
	*Enterococcus faecalis* DSMZ 12956 *Enterococcus faecium* DSMZ 13590 *Enterococcus flavescens* DSMZ 7370 *Enterococcus casseliflavus* DSMZ 20680 *Enterococcus gallinarum* DSMZ 20628	DSMZ
	*Listeria monocytogenes* L7946 *Listeria monocytogenes* L7947	McLauchlin et al. [16]
Gram-negative	*Acinetobacter baumannii* R *Acinetobacter baumannii* S-1 *Acinetobacter baumannii* S-2 *Acinetobacter calcoaceticus* R *Acinetobacter calcoaceticus* S *Klebsiella pneumoniae* *Proteus mirabilis* *Proteus vulgaris* *Pseudomonas aeruginosa* *Salmonella* Braenderup *Salmonella* Enteritidis *Salmonella* Typhimurium *Yersinia enterocolitica*	ESB culture collection
	*Escherichia coli* ATCC 25922	ATCC
	*Yersinia enterocolitica* NCTC 10406	NCTC
Yeasts	*Candida albicans* *Saccharomyces cerevisiae*	ESB

ESB: culture collection of *Escola Superior de Biotecnologia*; DSMZ: German Collection of Microorganisms and Cell Cultures; ATCC: American Type Culture Collection; NCTC: National Collection of Types Cultures—Culture Collection of Public Health England. S—Sensitive to several tested antibiotics; R—Resistant to several antibiotics

**Table 2:** Minimum inhibitory concentrations (MIC; μg/mL) of seven antibiotics for seven lactic acid bacteria (LAB) isolates.

	Amp	Gen	Kan	Str	Ery	Chl	Tet
**R23**	0.5	≤4	≤16	n.r.	≤0,5	≤2	≤4
**Q42**	1	≤4	≤16	≤32	≤0,5	≤2	≤4
**Q43**	2	≤4	≤16	≤32	≤0,5	≤2	≤4
**CFF4**	2	≤4	≤16	≤32	≤0,5	≤2	≤4
**CFF5**	2	≤4	≤16	≤32	≤0,5	≤2	≤4
**CFF51**	1	≤4	≤16	≤32	≤0,5	≤2	≤4
**CFF202**	1	≤4	≤16	≤32	≤0,5	≤2	≤4

Amp—ampicillin; Gent—gentamicin; Kan—kanamycin; Str—streptomycin; Ery—erythromycin; Chl—chloramphenicol; Tet—tetracycline; n.r.—not required.

**Table 3:** Survival of seven LAB isolates through simulated gastrointestinal (GI) tract conditions.

	Log CFU/mL (*)		
Isolate	0 min	60 min (#)	120 min (†)
*L. plantarum* R23	6.82 ± 0.06^Aa^	4.49 ± 0.04^Ba^	4.56 ± 0.05^Ba^
*P. pentosaceus* Q42	7.16 ± 0.02^Aa^	6.82 ± 0.06^Ab^	6.64 ± 0.09^Ab^
*P. pentosaceus* Q43	6.87 ± 0.20^Aa^	7.18 ± 0.20^Ab^	6.65 ± 0.09^Ab^
*P. pentosaceus* CFF4	7.25 ± 0.12^Aa^	7.12 ± 0.03^Ab^	6.90 ± 0.22^Abc^
*P. pentosaceus* CFF5	7.25 ± 0.03^Aa^	7.02 ± 0.17^Ab^	7.17 ± 0.13^Ac^
*P. pentosaceus* CFF51	7.06 ± 0.27^Aa^	7.30 ± 0.19^Ab^	7.04 ± 0.05^Abc^
*P. pentosaceus* CFF202	7.12 ± 0.07^Aa^	7.07 ± 0.16^Ab^	6.87 ± 0.06^Abc^

(*) Survival is represented as the mean of the log CFU/mL ± the standard error of the mean. (**#**) Survival after exposure to pH 2.5 in the presence of pepsin. (**†**) Survival after exposure to pH 2.5 in the presences of pepsin and subsequent exposure to bile salts at pH 7.0. Equivalent capital letters, per line, mean no significant differences between each condition (*p* > 0.05) Equivalent lowercase letters, by column, mean no significant differences between isolates at each condition (*p* > 0.05).

## Supplementary Materials

The following are available online at https://www.mdpi.com/2076-2607/8/3/393/s1, Figure S1: Phylogenetic relationship of the partial 16S rRNA gene sequences representing the isolates used in this study and of sequences obtained from the Genbank database. The 16S rRNA gene sequences from *Ferroplasma acidiphilum* were used as outgroup. Trees were constructed using the neighbour-joining method, using the MEGA-7 software. The scale bar indicates 0.1 nucleotide substitution per sequence position. Table S1: Origin of LAB isolates used in this study.

## References

[1] Food and Agriculture Organization/World Health Organization (2002). “Guidelines for the Evaluation of Probiotics in Foods”, Report of a Joint FAO/WHO Working Group on Drafting Guidelines for the Evaluation of Probiotics in Food.

[2] Amara A.A., Shibl A. (2015). Role of probiotics in health improvement, infection control and disease treatment and management. Saudi Pharm. J..

[3] Sathyabama S., Vijayabharathi R., Devi P.B., Kumar M.R., Priyadarisini V.B. (2012). Screening for probiotic properties of strains isolated from feces of various human groups. J. Microbiol..

[4] Hill C., Guarner F., Reid G., Gibson G.R., Merenstein D.J., Pot B., Morelli L., Canani R.B., Flint H.J., Salminen S. (2014). The international scientific association for probiotics and prebiotics consensus statement on the scope and appropriate use of the term probiotic. Nat. Rev. Gastroenterol. Hepatol..

[5] Kim S.-K., Guevarra R.B., Kim Y.-T., Kwon J., Kim H., Cho J.H., Kim H.B., Lee J.-H. (2019). Role of probiotics in human gut microbiome-associated diseases. J. Microbiol. Biotechnol..

[6] Jia F.-F., Zhang L.-J., Pang X.-H., Gu X.-X., Abdelazez A., Liang Y., Sun S.-R., Meng X.-C. (2017). Complete genome sequence of bacteriocin-producing *Lactobacillus plantarum* KLDS1. 0391, a probiotic strain with gastrointestinal tract resistance and adhesion to the intestinal epithelial cells. Genomics.

[7] Zmora N., Zilberman-Schapira G., Suez J., Mor U., Dori-Bachash M., Bashiardes S., Kotler E., Zur M., Regev-Lehavi D., Brik R.B. (2018). Personalized gut mucosal colonization resistance to empiric probiotics is associated with unique host and microbiome features. Cell.

[8] Zhao W., Liu Y., Latta M., Ma W., Wu Z., Chen P. (2019). Probiotics database: A potential source of fermented foods. Int. J. Food Prop..

[9] Sanders M.E., Merenstein D., Merrifield C.A., Hutkins R. (2018). Probiotics for human use. Nut. Bull..

[10] World Gastroenterology Organisation Global Guidelines Probiotics and prebiotics. https://www.google.com.hk/url?sa=t&rct=j&q=&esrc=s&source=web&cd=4&ved=2ahUKEwiqjdKgyZLoAhWTOnAKHdO5AdMQFjADegQIARAB&url=https%3A%2F%2Fwww.worldgastroenterology.org%2FUserFiles%2Ffile%2Fguidelines%2Fprobiotics-and-prebiotics-english-2017.pdf&usg=AOvVaw3DKlMGBkSgn_QTY7o_5VAX.

[11] Zommiti M., Bouffartigues E., Maillot O., Barreau M., Szunerits S., Sebei K., Feuilloley M., Connil N., Ferchichi M. (2018). In vitro assessment of the probiotic properties and bacteriocinogenic potential of *Pediococcus pentosaceus* MZF16 isolated from artisanal Tunisian meat “dried ossban”. Front. Microbiol..

[12] Barbosa J., Borges S., Teixeira P. (2015). *Pediococcus acidilactici* as a potential probiotic to be used in food industry. Int. J. Food Sci. Technol..

[13] Todorov S.D., Holzapfel W., Nero L.A. (2016). In vitro evaluation of beneficial properties of bacteriocinogenic *Lactobacillus plantarum* ST8Sh. Probiotics Antimicrob. Proteins.

[14] Moreno I., Marasca E.T.G., de Sá P.B.Z.R., de Souza Moitinho J., Marquezini M.G., Alves M.R.C., Bromberg R. (2018). Evaluation of probiotic potential of bacteriocinogenic lactic acid bacteria strains isolated from meat products. Probiotics Antimicrob. Proteins.

[15] Barbosa J., Borges S., Teixeira P. (2014). Selection of potential probiotic *Enterococcus faecium* isolated from Portuguese fermented food. Int. J. Food Microbiol..

[16] McLauchlin J., Hampton M.D., Shah S., Threlfall E.J., Wieneke A.A., Curtis G.D.W. (1997). Subtyping of *Listeria monocytogenes* on the basis of plasmid profiles and arsenic and cadmium susceptibility. J. Appl. Microbiol..

[17] Albano H., Pinho C., Leite D., Barbosa J., Silva J., Carneiro L., Magalhães R., Hogg T., Teixeira P. (2009). Evaluation of a bacteriocin-producing strain of *Pediococcus acidilactici* as a biopreservative for ‘‘Alheira”, a fermented meat sausage. Food Control.

[18] Vaz-Moreira I., Faria C., Lopes A.R., Svensson L., Falsen E., Moore E.R., Ferreira A.C., Nunes O.C., Manaia C.M. (2009). *Sphingobium vermicomposti* sp. nov., isolated from vermicompost. Int. J. Syst. Evol. Microbiol..

[19] Altschul S.F., Madden T.L., Shaffer A.A., Zhang J., Zhang Z., Miller W., Lipman D.J. (1997). Gapped BLAST and PSI-BLAST: A new generation of protein database search programs. Nucleic Acids Res..

[20] Treangen T.J., Ondov B.D., Koren S., Phillippy A.M. (2014). The Harvest suite for rapid core-genome alignment and visualization of thousands of intraspecific microbial genomes. Genome Biol..

[21] Bover-Cid S., Holzapfel W.H. (1999). Improved screening produce for biogenic amine production by lactic acid bacteria. Int. J. Food Microbiol..

[22] Tiago I., Teixeira I., Silva S., Chung P., Verıssimo A., Manaia C.M. (2004). Metabolic and genetic diversity of mesophilic and thermophilic bacteria isolated from composted municipal sludge on poly-e-caprolactones. Curr. Microbiol..

[23] Ben Omar N., Castro A., Lucas R., Abriouel H., Yousif N.M.K., Franz C.M.A.P., Holzapfel W.H., Rubén P.-P., Martínez-Canãmero M., Gálvez A. (2004). Functional and safety aspects of enterococci isolated from different Spanish foods. Syst. Appl. Microbiol..

[24] Barbosa J., Gibbs P.A., Teixeira P. (2010). Virulence factors among enterococci isolated from traditional fermented meat products produced in the North of Portugal. Food Control.

[25] European Food Safety Authority (2012). Guidance on the assessment of bacterial susceptibility to antimicrobials of human and veterinary importance by EFSA Panel on Additives and Products or Substances used in Animal Feed (FEEDAP). EFSA J..

[26] Clinical and Laboratory Standards Institute (2012). Performance Standards for Antimicrobial Susceptibility Testing.

[27] Miles A.A., Misra S.S. (1938). The estimation of the bactericidal power of blood. J. Hyg..

[28] Barbosa J., Borges S., Magalhães R., Ferreira V., Santos I., Silva J., Almeida G., Gibbs P., Teixeira P. (2012). Behaviour of *Listeria monocytogenes* isolates through gastro-intestinal tract passage simulation, before and after two sub-lethal stresses. Food Microbiol..

[29] Guglielmotti D.M., Marcó M.B., Golowczyc M., Reinheimer J.A., del Quiberoni A.A.L. (2007). Probiotic potential of *Lactobacillus delbrueckii* strains and their phage resistant mutants. Int. Dairy J..

[30] Tsai C., Lin P., Hsieh Y., Zhang Z.Y., Wu H.C., Huang C.C. (2014). Cholesterol-lowering potentials of lactic acid bacteria based on bile-salt hydrolase activity and effect of potent strains on cholesterol metabolism in vitro and in vivo. Sci. World J..

[31] Peng C., Borges S., Magalhães R., Carvalheira A., Ferreira V., Casquete R., Teixeira P. (2017). Characterization of anti-listerial bacteriocin produced by lactic acid bacteria isolated from traditional fermented foods from Cambodia. Int. Food Res. J..

[32] Collins M.D., Rodrigues U.M., Ash C., Aguirre M., Farrow J.A.E., Martinez-Murcia A., Phillips B.A., Williams A.M., Wallbanks S. (1991). Phylogenetic analysis of the genus *Lactobacillus* and related lactic acid bacteria as determined by reverse transcriptase sequencing of 16S rRNA. FEMS Microbiol. Lett..

[33] Albano H., Todorov S.D., van Reenen C.A., Hogg T., Dicks L.M., Teixeira P. (2007). Characterization of two bacteriocins produced by *Pediococcus acidilactici* isolated from “Alheira”, a fermented sausage traditionally produced in Portugal. Int. J. Food Microbiol..

[34] Sadishkumar V., Kolanchiammal R., Jeevaratnam K. (2015). The effect of Piper betle leaves on lacto-fermentation of idli batter, characterization and applicability of potent isolates in soymilk fermentation. Food Sci. Biotechnol..

[35] Abushelaibi A., Al-Mahadin S., El-Tarabily K., Shah N.P., Ayyash M. (2017). Characterization of potential probiotic lactic acid bacteria isolated from camel milk. LWT—Food Sci. Technol..

[36] Hernández-Alcántara A.M., Wacher C., Llamas M.G., López P., Pérez-Chabela M.L. (2018). Probiotic properties and stress response of thermotolerant lactic acid bacteria isolated from cooked meat products. LWT—Food Sci. Technol..

[37] Mulaw G., Tessema T.S., Muleta D., Tesfaye A. (2019). In vitro evaluation of probiotic properties of lactic acid bacteria isolated from some traditionally fermented Ethiopian food products. Int. J. Microbiol..

[38] Muñoz-Atienza E., Gómez-Sala B., Araújo C., Campanero C., del Campo R., Hernández P.E., Herranz C., Cintas L.M. (2013). Antimicrobial activity, antibiotic susceptibility and virulence factors of lactic acid bacteria of aquatic origin intended for use as probiotics in aquaculture. BMC Microbiol..

[39] Behera S.S., Ray R.C., Zdolec N. (2018). *Lactobacillus plantarum* with functional properties: An approach to increase safety and shelf-life of fermented foods. BioMed. Res. Int..

[40] Rozman V., Lorbeg P.M., Accetto T., Matijašić B.B. (2020). Characterization of antimicrobial resistance in lactobacilli and bifidobacteria used as probiotics or starter cultures based on integration of phenotypic and in silico data. Int. J. Food Microbiol..

[41] European Food Safety Authority (2005). Opinion of the Scientific Committee on a request from EFSA related to a generic approach to the safety assessment by EFSA of microorganisms used in food/feed and the production of food/feed additives. EFSA J..

[42] European Food Safety Authority (2005). Opinion of the Scientific Panel on Additives and Products or Substances used in Animal Feed on the updating of the criteria used in the assessment of bacteria for resistance to antibiotics of human or veterinary importance. EFSA J..

[43] Vera-Pingitore E., Jimenez M., Dallagnol A., Belfiore C., Fontana C., Fontana P., von Wright A., Vignolo G., Plumed-Ferrer C. (2016). Screening and characterization of potential probiotic and starter bacteria for plant fermentations. LWT—Food Sci. Technol..

[44] García-Hernández Y., Pérez-Sánchez T., Boucourt R., Balcázar J., Nicoli J., Moreira-Silva J., Rodríguez Z., Fuertes H., Nuñez O., Albelo N. (2016). Isolation, characterization and evaluation of probiotic lactic acid bacteria for potential use in animal production. Res. Vet. Sci..

[45] Damodharan K., Lee Y.S., Palaniyandi S.A., Yang S.H., Suh J.-W. (2015). Preliminary probiotic and technological characterization of *Pediococcus pentosaceus* strain KID7 and in vivo assessment of its cholesterol-lowering activity. Front. Microbiol..

[46] Han Q., Kong B., Chen Q., Sun F., Zhang H. (2017). In vitro comparison of probiotic properties of lactic acid bacteria isolated from Harbin dry sausages and selected probiotics. J. Funct. Foods.

[47] Duary R.K., Rajput Y.S., Batish V.K., Grover S. (2011). Assessing the adhesion of putative indigenous probiotic lactobacilli to human colonic epithelial cells. Indian J. Med. Res..

[48] Begley M., Hill C., Gahan C.G.M. (2006). Bile Salt Hydrolase Activity in Probiotics. Appl. Environ. Microbiol..

[49] Noriega L., Cuevas I., Margolles A., de los Reyes-Gavilán C.G. (2006). Deconjugation and bile salts hydrolase activity by *Bifidobacterium* strains with acquired resistance to bile. Int. Dairy J..

