# Supplementary material for: Screening of Bacteriocinogenic Lactic Acid Bacteria and Their Characterization as Potential Probiotics
Source: Microorganisms. 2020 Mar 11;8(3):393. doi: 10.3390/microorganisms8030393 (PMC7142618; doi:10.3390/microorganisms8030393)
Supplement: Supplementary file 1 [file microorganisms-08-00393-s001.zip › microorganisms-721752-supplementary/Supplementary files/Figure S1.pdf]

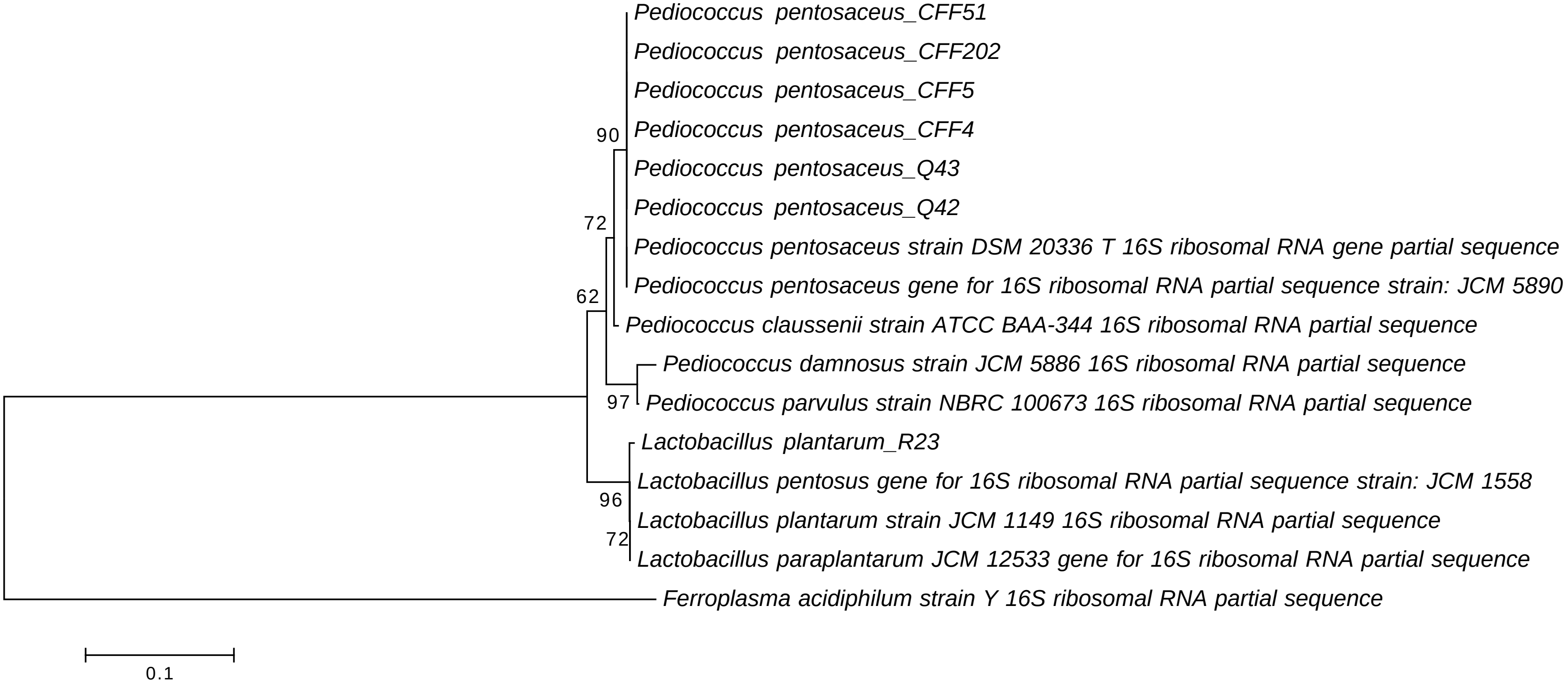
Pediococcus pentosaceus_CFF51
Pediococcus pentosaceus_CFF202
Pediococcus pentosaceus_CFF5
Pediococcus pentosaceus_CFF4
Pediococcus pentosaceus_Q43
Pediococcus pentosaceus_Q42
Pediococcus pentosaceus strain DSM 20336 T 16S ribosomal RNA gene partial sequence
Pediococcus pentosaceus gene for 16S ribosomal RNA partial sequence strain: JCM 5890
Pediococcus claussenii strain ATCC BAA-344 16S ribosomal RNA partial sequence
Pediococcus damnosus strain JCM 5886 16S ribosomal RNA partial sequence
Pediococcus parvulus strain NBRC 100673 16S ribosomal RNA partial sequence
Lactobacillus plantarum_R23
Lactobacillus pentosus gene for 16S ribosomal RNA partial sequence strain: JCM 1558
Lactobacillus plantarum strain JCM 1149 16S ribosomal RNA partial sequence
Lactobacillus paraplantarum JCM 12533 gene for 16S ribosomal RNA partial sequence
Ferroplasma acidiphilum strain Y 16S ribosomal RNA partial sequence
90
72
62
97
96
72
0.1
