# Supplementary material for: Screening of Bacteriocinogenic Lactic Acid Bacteria and Their Characterization as Potential Probiotics
Source: Microorganisms. 2020 Mar 11;8(3):393. doi: 10.3390/microorganisms8030393 (PMC7142618; doi:10.3390/microorganisms8030393)
Supplement: Supplementary file 1 [file microorganisms-08-00393-s001.zip › microorganisms-721752-supplementary/Supplementary files/Table S1.pdf]

**Table S1.** Origin of LAB isolates used in this study

| LAB | Origin | LAB | Origin | LAB | Origin | LAB | Origin |
|---|---|---|---|---|---|---|---|
| A1.1 | Fermented sausage | A9.9 | Fermented sausage | A19.7 | Fermented sausage | A31.3 | Fermented sausage |
| A1.2 | Fermented sausage | A10.1 | Fermented sausage | A19.8 | Fermented sausage | A31.4 | Fermented sausage |
| A1.3 | Fermented sausage | A10.2 | Fermented sausage | A20.1 | Fermented sausage | A31.5 | Fermented sausage |
| A1.4 | Fermented sausage | A10.3 | Fermented sausage | A20.2 | Fermented sausage | A31.6 | Fermented sausage |
| A1.5 | Fermented sausage | A10.4 | Fermented sausage | A20.3 | Fermented sausage | A32.1 | Fermented sausage |
| A1.6 | Fermented sausage | A10.5 | Fermented sausage | A20.4 | Fermented sausage | A32.2 | Fermented sausage |
| A1.7 | Fermented sausage | A10.6 | Fermented sausage | A20.5 | Fermented sausage | A32.3 | Fermented sausage |
| A2.1 | Fermented sausage | A10.7 | Fermented sausage | A21.1 | Fermented sausage | A33.1 | Fermented sausage |
| A2.2 | Fermented sausage | A10.8 | Fermented sausage | A21.2 | Fermented sausage | A33.2 | Fermented sausage |
| A2.3 | Fermented sausage | A10.9 | Fermented sausage | A21.3 | Fermented sausage | A33.3 | Fermented sausage |
| A2.4 | Fermented sausage | A10.10 | Fermented sausage | A21.4 | Fermented sausage | A33.4 | Fermented sausage |
| A2.5 | Fermented sausage | A10.11 | Fermented sausage | A22.1 | Fermented sausage | A33.5 | Fermented sausage |
| A3.1 | Fermented sausage | A11.1 | Fermented sausage | A22.2 | Fermented sausage | A33.6 | Fermented sausage |
| A3.2 | Fermented sausage | A11.2 | Fermented sausage | A22.3 | Fermented sausage | A33.7 | Fermented sausage |
| A3.3 | Fermented sausage | A11.3 | Fermented sausage | A22.4 | Fermented sausage | A34.1 | Fermented sausage |
| A3.4 | Fermented sausage | A11.4 | Fermented sausage | A22.5 | Fermented sausage | A34.2 | Fermented sausage |
| A3.5 | Fermented sausage | A11.5 | Fermented sausage | A22.6 | Fermented sausage | A34.3 | Fermented sausage |
| A3.6 | Fermented sausage | A11.6 | Fermented sausage | A23.1 | Fermented sausage | A34.4 | Fermented sausage |
| A3.7 | Fermented sausage | A11.7 | Fermented sausage | A23.2 | Fermented sausage | A34.5 | Fermented sausage |
| A4.1 | Fermented sausage | A11.8 | Fermented sausage | A23.3 | Fermented sausage | A35.1 | Fermented sausage |
| A4.2 | Fermented sausage | A12.1 | Fermented sausage | A23.4 | Fermented sausage | A35.2 | Fermented sausage |
| A4.3 | Fermented sausage | A12.2 | Fermented sausage | A23.5 | Fermented sausage | A35.3 | Fermented sausage |
| A4.4 | Fermented sausage | A12.3 | Fermented sausage | A23.6 | Fermented sausage | A35.4 | Fermented sausage |
| A4.5 | Fermented sausage | A12.4 | Fermented sausage | A23.7 | Fermented sausage | A35.5 | Fermented sausage |
| A4.6 | Fermented sausage | A12.5 | Fermented sausage | A24.1 | Fermented sausage | A35.6 | Fermented sausage |
| A4.7 | Fermented sausage | A12.6 | Fermented sausage | A24.2 | Fermented sausage | A36.1 | Fermented sausage |
| A4.8 | Fermented sausage | A12.7 | Fermented sausage | A24.3 | Fermented sausage | A36.2 | Fermented sausage |
| A4.9 | Fermented sausage | A12.8 | Fermented sausage | A24.4 | Fermented sausage | A36.3 | Fermented sausage |
| A4.10 | Fermented sausage | A12.9 | Fermented sausage | A24.5 | Fermented sausage | A36.4 | Fermented sausage |
| A4.11 | Fermented sausage | A12.10 | Fermented sausage | A24.6 | Fermented sausage | A36.5 | Fermented sausage |
| A5.1 | Fermented sausage | A12.11 | Fermented sausage | A24.5 | Fermented sausage | A36.6 | Fermented sausage |
| A5.2 | Fermented sausage | A12.12 | Fermented sausage | A25.1 | Fermented sausage | A36.7 | Fermented sausage |
| A5.3 | Fermented sausage | A13.1 | Fermented sausage | A25.2 | Fermented sausage | A36.8 | Fermented sausage |
| A5.4 | Fermented sausage | A13.2 | Fermented sausage | A25.3 | Fermented sausage | A36.9 | Fermented sausage |
| A5.5 | Fermented sausage | A13.3 | Fermented sausage | A25.4 | Fermented sausage | A36.10 | Fermented sausage |
| A5.6 | Fermented sausage | A13.4 | Fermented sausage | A25.5 | Fermented sausage | K34 | Fermented sausage |
| A5.7 | Fermented sausage | A13.5 | Fermented sausage | A25.6 | Fermented sausage | Q42 | Cheese |
| A5.8 | Fermented sausage | A13.6 | Fermented sausage | A26.1 | Fermented sausage | Q43 | Cheese |
| A5.9 | Fermented sausage | A13.7 | Fermented sausage | A26.2 | Fermented sausage | Q43 | Cheese |
| A5.10 | Fermented sausage | A13.8 | Fermented sausage | A26.3 | Fermented sausage | DT016 | Lettuce |
| A6.1 | Fermented sausage | A14.1 | Fermented sausage | A26.4 | Fermented sausage | SB83 | Seafood |
| A6.2 | Fermented sausage | A14.2 | Fermented sausage | A26.5 | Fermented sausage | ALP7 | Shellfish |
| A6.3 | Fermented sausage | A14.3 | Fermented sausage | A26.6 | Fermented sausage | R23 | Arugula |
| A6.4 | Fermented sausage | A15.1 | Fermented sausage | A26.7 | Fermented sausage | CFF4 | Cambodian fermented fish |
| A7.1 | Fermented sausage | A15.2 | Fermented sausage | A27.1 | Fermented sausage | CFF5 | Cambodian fermented fish |
| A7.2 | Fermented sausage | A15.3 | Fermented sausage | A27.2 | Fermented sausage | CFF51 | Cambodian fermented fish |
| A7.3 | Fermented sausage | A15.4 | Fermented sausage | A27.3 | Fermented sausage | CFF202 | Cambodian fermented fish |
| A7.4 | Fermented sausage | A15.5 | Fermented sausage | A27.4 | Fermented sausage | ST153 | Smoked salmon |
| A7.5 | Fermented sausage | A16.1 | Fermented sausage | A27.5 | Fermented sausage | SS100.2 | Smoked salmon |
| A7.6 | Fermented sausage | A16.2 | Fermented sausage | A28.1 | Fermented sausage | SS102.1 | Smoked salmon |
| A7.7 | Fermented sausage | A16.3 | Fermented sausage | A28.2 | Fermented sausage | SS104.1 | Smoked salmon |
| A7.8 | Fermented sausage | A16.4 | Fermented sausage | A28.3 | Fermented sausage | SS107.1 | Smoked salmon |
| A7.9 | Fermented sausage | A16.5 | Fermented sausage | A28.4 | Fermented sausage | SS107.2 | Smoked salmon |
| A7.10 | Fermented sausage | A16.6 | Fermented sausage | A28.5 | Fermented sausage | SS110 | Smoked salmon |
| A7.11 | Fermented sausage | A16.7 | Fermented sausage | A28.6 | Fermented sausage | SS114 | Smoked salmon |
| A8.1 | Fermented sausage | A16.8 | Fermented sausage | A29.1 | Fermented sausage | SS117 | Smoked salmon |
| A8.2 | Fermented sausage | A17.1 | Fermented sausage | A29.2 | Fermented sausage | SS118 | Smoked salmon |
| A8.3 | Fermented sausage | A17.2 | Fermented sausage | A29.3 | Fermented sausage | SS121 | Smoked salmon |
| A8.4 | Fermented sausage | A18.1 | Fermented sausage | A29.4 | Fermented sausage | SS126.2 | Smoked salmon |
| A8.5 | Fermented sausage | A18.2 | Fermented sausage | A29.5 | Fermented sausage | SS127 | Smoked salmon |
| A8.6 | Fermented sausage | A18.3 | Fermented sausage | A29.6 | Fermented sausage | SS128 | Smoked salmon |
| A8.7 | Fermented sausage | A18.4 | Fermented sausage | A29.7 | Fermented sausage | SS139 | Smoked salmon |
| A9.1 | Fermented sausage | A18.5 | Fermented sausage | A29.8 | Fermented sausage | SS144 | Smoked salmon |
| A9.2 | Fermented sausage | A18.6 | Fermented sausage | A30.1 | Fermented sausage | SS148 | Smoked salmon |
| A9.3 | Fermented sausage | A19.1 | Fermented sausage | A30.2 | Fermented sausage | SS149 | Smoked salmon |
| A9.4 | Fermented sausage | A19.2 | Fermented sausage | A30.3 | Fermented sausage | SS165 | Smoked salmon |
| A9.5 | Fermented sausage | A19.3 | Fermented sausage | A30.4 | Fermented sausage | SS167 | Smoked salmon |
| A9.6 | Fermented sausage | A19.4 | Fermented sausage | A30.5 | Fermented sausage | SS168 | Smoked salmon |
| A9.7 | Fermented sausage | A19.5 | Fermented sausage | A31.1 | Fermented sausage | SS170 | Smoked salmon |
| A9.8 | Fermented sausage | A19.6 | Fermented sausage | A31.2 | Fermented sausage | SS195 | Smoked salmon |
